# The Role of Akt2 in the Protective Effect of Fenofibrate against Diabetic Nephropathy

**DOI:** 10.7150/ijbs.40643

**Published:** 2020-01-01

**Authors:** Yanli Cheng, Xiaoyu Zhang, Fuzhe Ma, Weixia Sun, Wanning Wang, Jinyu Yu, Yue Shi, Lu Cai, Zhonggao Xu

**Affiliations:** 1 Department of Nephrology, The First Hospital of Jilin University, Changchun, Jilin, 130021, China;; 2 Department of Gastrointestinal and Colorectal Surgery, China-Japan Union Hospital of Jilin University, Changchun, Jilin, 130033, China;; 3 Department of Microbiology and Immunology, Changchun University of Chinese Medicine, Changchun, Jilin, 130117, China;; 4 Pediatric Research Institute, Departments of Pediatrics, Radiation Oncology, Pharmacology and Toxicology, University of Louisville, Louisville, KY, 40202, USA.

**Keywords:** diabetic nephropathy, Akt2, fibroblast growth factor 21, nuclear factor erythroid 2-related factor 2, peroxisome proliferator-activated receptor α agonist.

## Abstract

Fenofibrate (FF) protects against diabetic nephropathy (DN) in type 1 diabetic (T1D) mice by upregulating the expression of fibroblast growth factor 21 (FGF21), leading to the activation of the Akt-mediated Nrf2 antioxidant pathways. Here, we examined which isoforms of Akt contribute to FF activation of FGF21-mediated renal protection by examining the phosphorylation and expression of three isoforms, Akt1, Akt2, and Akt3. T1D induced by a single intraperitoneal dose of streptozotocin (STZ) resulted in reduced phosphorylation of one isoform, Akt2, but FF treatment increased renal Akt2 phosphorylation in these and normal mice, suggesting a potential and specific role for renal Akt2 in FF protection against T1D. This was further confirmed using *in vitro* cultured HK-2 human kidney tubule cells exposed to high glucose (HG) with siRNA silencing of the *Akt2* gene and STZ-induced diabetic *Akt2*-knockout mice with and without 3-month FF treatment. In normal HK-2 cells exposed to HG for 24 hours, FF completely prevented cell death, reduced total Akt expression and glycogen synthase kinase (GSK)-3β phosphorylation, increased nuclear accumulation of Fyn, and reduced nuclear Nrf2 levels. These positive effects of FF were partially abolished by silencing *Akt2* expression. Similarly, FF abolished T1D-induced renal oxidative stress, inflammation, and renal dysfunction in wild-type mice, but was only partially effective in Akt2-KO mice. Furthermore, FF treatment stimulated phosphorylation of AMPKα, an important lipid metabolism mediator, which in parallel with Akt2 plays an important role in FF protection against HG-induced HK-2 cells oxidative stress and damage. These results suggest that FF protects against DN through FGF21 to activate both Akt2/GSK-3β/Fyn/Nrf2 antioxidants and the AMPK pathway. Therefore, FF could be repurposed for the prevention of DN in T1D patients.

## Introduction

Diabetic nephropathy (DN) is one of the most serious complications of diabetes mellitus 1, which is generally characterized by glomerular basement membrane thickening, mesangial stromal hyperplasia, interstitial fibrosis, and the apoptosis of podocytes and renal tubular epithelial cells, eventually leading to greater proteinuria and renal failure 2, 3. It is thought that oxidative stress resulting from hyperglycemia is an important mechanism underpinning the development of diabetic complications 4, 5. Therefore, upregulation of multiple endogenous antioxidant genes and suppression of oxidative stress may provide an appropriate strategy for the prevention or treatment of diabetic complications 6, 7.

Accumulating evidence indicates that the activation of nuclear factor erythroid 2-related factor 2 (Nrf2), an important transcription factor that mediates oxidative stress resistance, is protective against renal injury 8, 9. Fenofibrate (FF) is a peroxisome proliferator-activated receptor-alpha (PPARα) agonist that is commonly used as a lipid-lowering medication 10. However, previous studies have shown that the protective effect of FF against DN can be independent of its lipid-lowering effect 11, 12. FF also has renal protective effects in a model of type 1 diabetes (T1D), which are achieved through activation of the transforming growth factor (TGF)-β1/smad3 and canonical Wnt pathways that ameliorate renal fibrosis 13, 14. However, the underlying mechanisms of the renal protective effects of FF in T1D have not been fully investigated. Fibroblast growth factor 21 (FGF21) is a direct target of PPARα that can protect against organ damage through multiple mechanisms and resist oxidative stress by upregulating Nrf2 15. FF directly regulates the synthesis and secretion of FGF21, a key regulator of glycolipid metabolism 16.

Previous studies have shown that the Akt signaling pathway mediates the effects of FGF21 on glycolipid metabolism 17, 18. Our previous study demonstrated that FF can prevent oxidative stress, cell apoptosis, inflammation, and fibrosis, reverse renal remodeling and insufficiency in mice with T1D, and upregulate the expression of Nrf2 19. In addition, we found that in mice with T1D treated with FF, FGF21 activated the Akt signaling pathway, because deletion of the FGF21 gene significantly reduced Akt expression and function 19. There are three isoforms of Akt: Akt1 (PKBα), Akt2 (PKBβ), and Akt3 (PKBγ), which each have their own physiologic functions. However, whether Akt activation and Nrf2 upregulation plays important roles in the effects of FF treatment on DN, which Akt isoforms up-regulated by FF provides protection on DN, whether its isoform is the only target in the process of FF protection on DN by mediating Nrf2 up-regulation induced by FGF21. All these issues are still remains to be fully established. Therefore, in the present study, we aimed to investigate the mechanism involved in the effect of FF to protect against DN, with an emphasis on determining which Akt isoform mediates the FGF21-induced upregulation of Nrf2 expression.

## Materials and methods

### Animals

Eight-week-old male *Akt2*-KO mice that were obtained by breeding homozygotes (*Akt2*^-/-^) with heterozygotes (*Akt2*^+/-^) purchased from the Jackson Laboratory (Bar Harbor, ME). Age- and sex-matched C57BL/6J mice were used in the present study. The mice were housed at 22°C on a 12:12 h light-dark cycle, with free access to standard rodent chow and tap water, in the Animal Experiment Center of the Basic Medical College of Jilin University. All animal procedures were approved by the Institutional Animal Care and Use Committee of the Jilin University.

### Steptozotocin-induced diabetes in Akt2-KO mice

As previously reported, a typical DN model was established by injecting intraperitoneally C57BL/6J mice with a single 150 mg/kg dose of streptozotocin (STZ, Sigma-Aldrich, St. Louis, MO, USA) dissolved in 0.1 M sodium citrate buffer (pH 4.5). Control animals were injected with the same volume of citrate buffer 20. Therefore, both *Akt2*-KO mice and their wild-type C57BL/6J counterparts were treated as described above, and three days after injection, hyperglycemia (blood glucose concentration ≥ 250 mg/dl) was detected in the STZ-injected mice. Both the diabetic and age-matched control mice were randomly allocated to two groups, which were treated with 100 mg/kg FF (Sigma-Aldrich) dissolved in 1% sodium carboxyl methylcellulose (Na-CMC) or 1% Na-CMC alone by gavage every other day for 3 months. After this, the mice were euthanized for the collection of blood, urine, and kidney tissue for protein, mRNA, and histopathologic analysis.

### Cell culture and treatment

Human renal tubular epithelial (HK-2) cells were cultured in Dulbecco's-modified Eagle's medium supplemented with 10% fetal bovine serum, 100 U/L penicillin, and 100 U/L streptomycin, in a humidified atmosphere of 5% CO_2_, at 37°C. The HK-2 cells were initially cultured in 5 mM glucose and made quiescent by incubation overnight in serum-free medium. After this, the cells were incubated in medium containing 30 mM glucose and/or FF and/or compound C (20 μM, an inhibitor of adenosine monophosphate-activated protein kinase [AMPK]) for 24 h.

### siRNA and expression plasmid transfection

*Akt2* siRNA was designed using a GeneScript siRNA design tool and the targeting sequences 5′-UGACUUCGACUAUCUCAAATT-3′ (forward) and 5′-UUUGAGAUAGUCGAAGUCATT-3′ (reverse) corresponding to the cDNA sequence between 450 and 468 bp. This test or control siRNA (GenePharma, Shanghai, China) were transfected into HK-2 cells using Lipofectamine^TM^ RNAiMAX (Invitrogen, CA, USA), according to the manufacturer's instructions.

### Apoptosis in cultured tubule cells

An apoptosis assay was performed after treatment using an Annexin V FITC/PI Apoptosis Detection Kit (BD Biosciences, Franklin Lakes, NJ, USA), following the manufacturer's protocol, and was then evaluated with an Accuri C6 flow cytometer (BD Biosciences, NJ, USA) and Cell Quest Pro Software (BD Biosciences, NJ, USA). Annexin V bound to phosphatidylserine (PS) in the cell membrane of viable apoptotic cells, and was identified by labeling with fluorescein isothiocyanate (FITC). The nuclei of non-viable apoptotic cells and dead cells were identified by propidium iodide (PI) staining. Thus, in combination, annexin V and PI can be used to distinguish early apoptotic cells and late apoptotic cells; therefore, we were able to analyze the extent of early apoptosis.

### Urine albumin-to-creatinine ratio

Spot urine samples (excluding the first urine of the morning) were collected from individual mice using clean Wide-Mouth Straight-Sided PMP Jars (Thermo Scientific, NY, USA) by bladder palpation. Urine samples were discarded if they were found to be contaminated with feces, food, or water, and the samples were kept frozen at -20°C until analysis 20. Urine albumin and creatinine concentrations were measured using kits from Bethyl Laboratories (Montgomery, TX, USA) and BioAssay Systems (Hayward, CA, USA), respectively, according to the manufacturer's instructions. Urinary albumin-to-creatinine ratio (UACR) was calculated to evaluate renal function, as UACR = urine albumin/urine creatinine (μg/mg).

### Renal histopathologic assessment and immunohistochemical staining

Kidney tissue was fixed in 10% formalin for 24 h, embedded in paraffin, and sectioned at 5 µm thickness for pathologic assessment and immunohistochemical (IHC) staining. The kidney sections were deparaffinized and rehydrated, and then stained with hematoxylin and eosin (H&E) to evaluate the histopathology. Periodic acid-Schiff (PAS) staining was used to visualize the renal glycogen content, as described previously 21. Renal fibrosis was visualized by Masson's staining for collagen, as described previously 20, 22, using a Sigma-Aldrich Trichrome Staining Kit. IHC staining with anti-FGF21 antibody (1:200 dilution, Antibody & Immunoassay Services, University of Hong Kong, China) was also performed. All the stained sections were examined using a Nikon Eclipse E600 microscopy system.

### Western blotting

Western blotting was performed as previously described 23. Kidney tissue and HK-2 cells were homogenized in RIPA lysis buffer (Santa Cruz Biotechnology) and then the nuclear fraction was isolated using a Nuclei Isolation Kit (Sigma-Aldrich), as previously described 24. After collection by centrifugation at 12,000 rpm at 4°C, the lysates and nuclear fractions were separated by 10% sodium dodecyl sulfate polyacrylamide gel electrophoresis (SDS-PAGE), and the proteins were transferred to nitrocellulose membranes (Bio-Rad, Hercules, CA, USA).

The membranes were blocked using 5% non-fat milk or 0.5% bovine serum albumin for 1 h, and then incubated overnight at 4°C with an antibody targeting one of the following: connective tissue growth factor (CTGF, 1:500 dilution), Nrf2 (1:1000 dilution), histone H3 (1:10,000 dilution), β-actin (1:3,000 dilution) (all purchased from Santa Cruz Biotechnology), phosphorylated Akt [*p*-Akt (Ser473)], total Akt1 (*t*-Akt1) and *p*-Akt1, *t*-Akt2 and *p*-Akt2, *t*-Akt3, *t*-GSK-3β and *p*-GSK-3β (Ser9), *p*-AMPKα (Thr172), *t*-AMPKα, Fyn (all purchased from Cell Signaling Technology and used at a 1:1,000 dilution), 4-hydroxy-2-nonenal (4-HNE, 1:4,000 dilution, Alpha Diagnostic International, San Antonio, TX, USA), tumor necrosis factor-alpha (TNF-α, 1:500 dilution, Abcam), or FGF21 (1:2,000 dilution, Antibody & Immunoassay Services, University of Hong Kong, China).

After three washes with Tris-buffered saline containing 0.05% Tween-20, membranes were incubated with the appropriate horseradish peroxidase (HRP)-conjugated secondary antibody (Santa Cruz Biotechnology) for 1 h at room temperature. Specific bands were visualized using an enhanced chemiluminescence kit (ECL, Thermo Fisher Scientific, Waltham, MA, USA). Densitometry was performed on the identified bands using Image Quant 5.2 software.

### Quantitative real-time PCR (qRT-PCR)

Total RNA was extracted using Trizol reagent (RNA STAT 60 Tel-Test Ambion, Austin, TX, USA) and a Nanodrop ND-1000 spectrophotometer was used to quantify RNA concentration and purity. First-strand complimentary DNA (cDNA) was synthesized from total RNA using an RNA PCR kit (Promega, Madison, WI, USA), according to the manufacturer's protocol. Reverse transcription was performed in a Mastercycler Gradient (Eppendorf, Hamburg, Germany) using 1 µg of total RNA in a 20 μL volume containing 4 μL 25 mM MgCl_2_, 4 μL AMV reverse transcriptase 5 × buffer, 2 μL dNTP, 0.5 μL RNase inhibitor, 1 μL of AMV reverse transcriptase, 1 μL of oligo (dT) primer, and nuclease-free water at 42°C for 50 min, followed by 95°C for 5 min. Primers for *NQO1* (Mm01253561), *HMOX1* (Mm00516005), and *ACTB* (Mm00607939) were purchased from Applied Biosystems (Carlsbad, CA, USA). qRT-PCR was performed in duplicate in 20 μL volumes containing 10 μL of TaqMan Universal PCR master mix, 9 μL of cDNA, and 1 μL of primer on an ABI 7500 Real-Time PCR system (Life Technologies Corp., Carlsbad, CA, USA). The comparative cycle time (Ct) method was used to determine the fold differences between the samples with respect to the amount of target present, which was normalized to *ACTB* as an endogenous reference, and relative to a calibrator (2^-△△Ct^).

### Statistical analysis

Data are presented as mean ± standard deviation (SD) (n ≥ 5). Comparisons were performed by one-way or two-way ANOVA for the different groups, followed by post-hoc pairwise repetitive comparisons using Tukey's test with GraphPad Prism version 6.0 (GraphPad Software, San Diego, CA) data analysis and graphing software.

## Results

### FF upregulates the phosphorylation of Akt2 in the kidney of T1D mice

**Figure 1:**
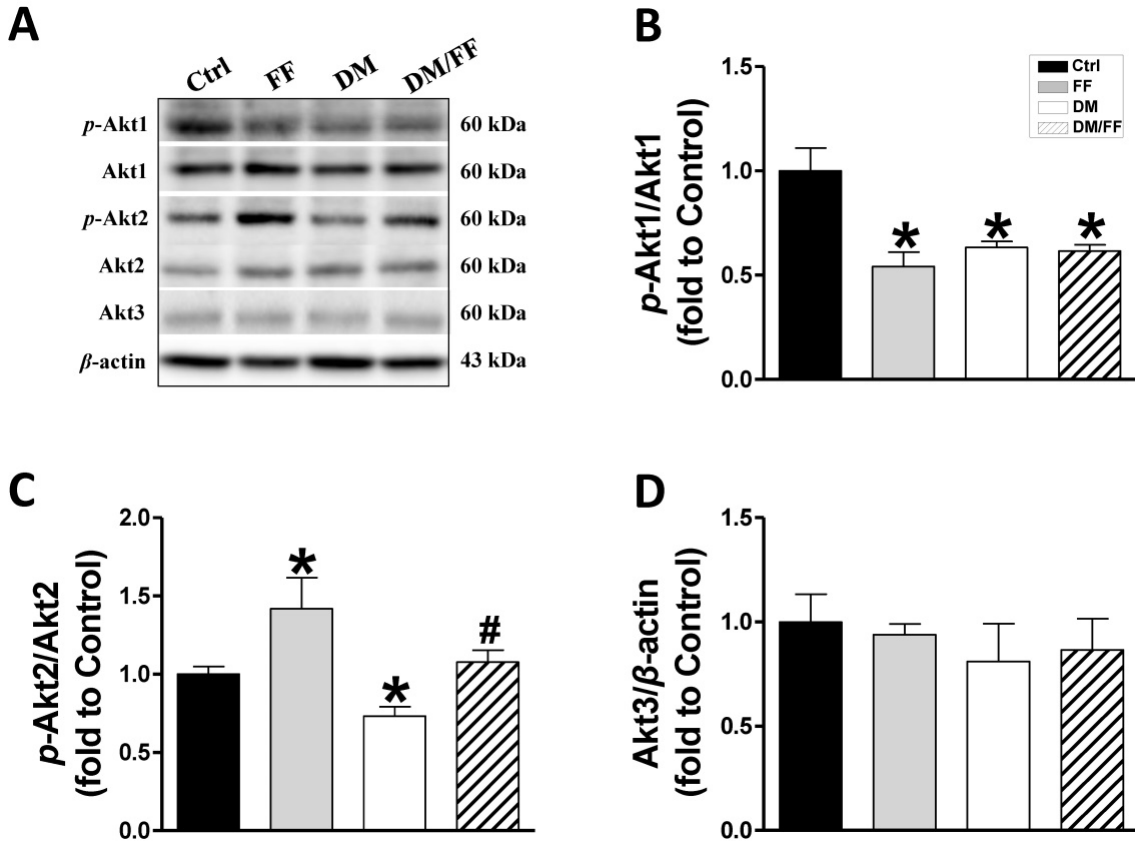
** Effects of the PPARα agonist fenofibrate (FF) on Akt isoforms in type 1 diabetic mice.** Diabetes was induced by a single intraperitoneal injection of streptozotocin (STZ, 150 mg/kg), and then mice were administered FF (100 mg/kg) or vehicle by gavage every other day for 3 months. Western blotting was used to measure total and phosphorylated Akt1 (A, B), total and phosphorylated Akt2 (A, C), and total Akt3 (A, D) expression. Data are presented as the mean ± SD (n≥5). *, *p* < 0.05 *vs*. control mice (Ctrl); #, *p* < 0.05 *vs*. diabetic mice (DM).

In our previous study, a renal UACR function test showed that UACR significantly increased in the third month of diabetes and that FF treatment significantly reduced this increase in UACR. Renal pathogenic alterations typically occurred in DM-induced nephropathy in diabetic mice, but not in FF-treated diabetic mice 19. Western blot analysis of renal tissues from the different animal groups showed that FF, diabetes, or DM/FF significantly affected Akt1, Akt2, and Akt3 protein expression. However, the phosphorylation levels of Akt1, in both FF-treated and streptozotocin-treated (T1D) mice were reduced, but the effects of FF and streptozotocin were not synergistic (Figure 1A, B). By contrast, FF treatment significantly increased renal Akt2 phosphorylation in normal and streptozotocin-treated (T1D) mice. (Figure 1A, C). This pilot study shows that Akt2 phosphorylation in the kidney increases significantly following a 3-month treatment with FF, and that this increase coincides with the amelioration of renal protection in FF-treated T1D-induced diabetes observed in the previous study.

### Akt2 silencing increases high glucose-induced human renal tubular epithelial (HK-2) cell apoptosis and causes a partial loss of FF-induced renal protection

**Figure 2:**
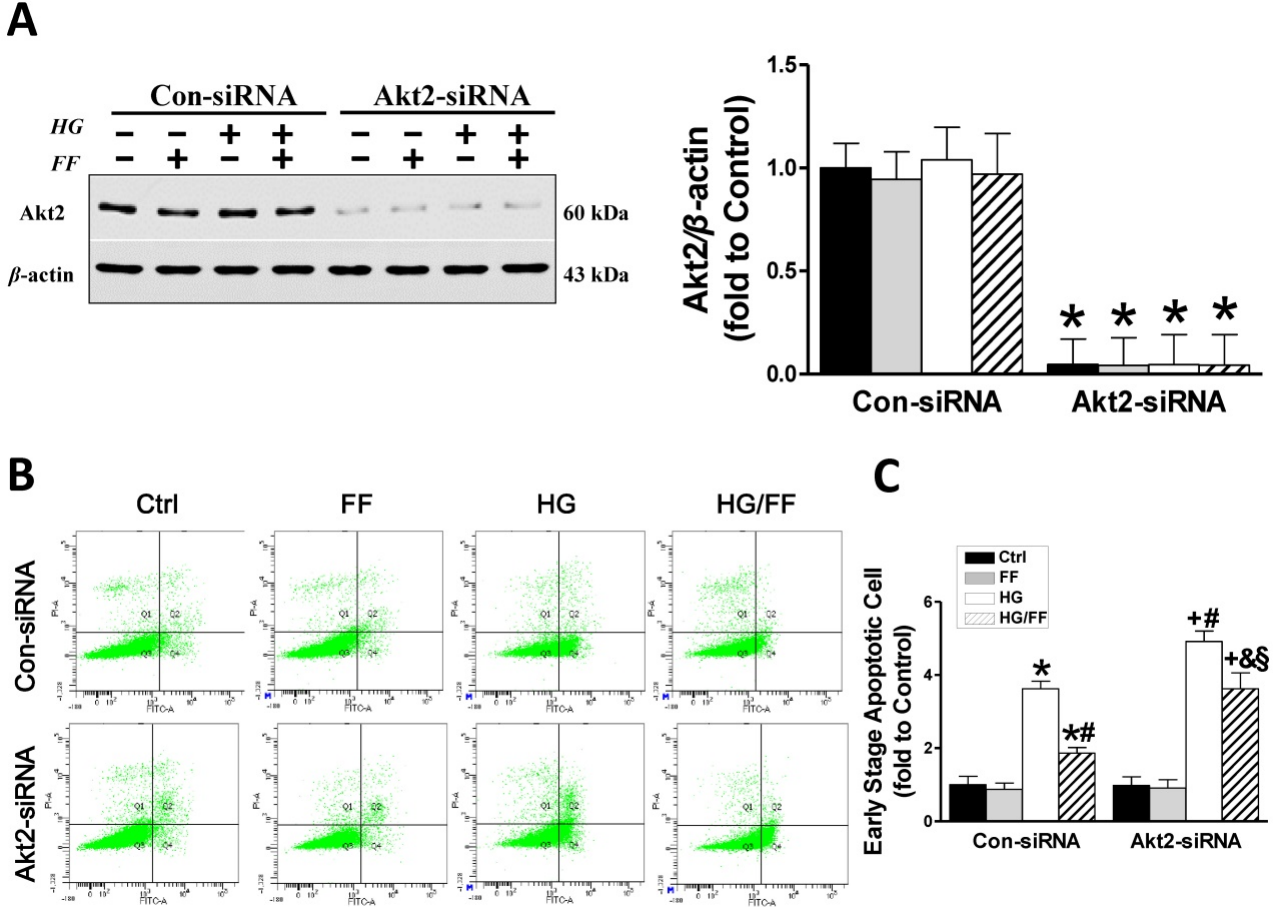
Akt2 silencing increases high glucose (HG)-induced human renal tubular epithelial (HK-2) cell apoptosis and reduces the renal protective effect of FF. Human renal tubular epithelial (HK-2) cells were treated with either control or *Akt2*-specific siRNA, and then the cells were treated with combinations of HG and FF for 24 h. Western blotting, an Annexin V FITC/PI Apoptosis Detection Kit, and an Accuri C6 flow cytometer and Cell Quest Pro Software were used to assess the outcomes. (A) Total Akt2 expression. (B) Apoptosis. Q1 represents dead cells, Q2 represents non-viable apoptotic cells, Q3 represents normal cells, and Q4 represents viable apoptotic cells. Quantitative analysis is shown in (C). Data were collected from at least three independent experiments and are presented as the mean ± SD (n≥5). *, *p* < 0.05 *vs*. the corresponding Con-siRNA/Ctrl; #, *p* < 0.05 *vs*. the corresponding Con-siRNA/HG; &, *p* < 0.05 *vs*. the corresponding Con-siRNA/HG/FF; +, *p* < 0.05 *vs*. the corresponding *Akt2*-siRNA/Ctrl; §, *p* < 0.05 *vs*. the corresponding *Akt2*-siRNA/HG.

To demonstrate that the increase in Akt2 phosphorylation after FF-treatment mediates FF prevention of T1D-induced renal pathogenesis, we established an *in vitro* assay using human renal tubular epithelial (HK-2) cells transfected with *Akt2*-specific siRNA (Akt2-siRNA) for 48 h to inhibit Akt2 expression, as well as with a control siRNA (Con-siRNA). Figure 2A shows that Akt2 expression was substantially reduced by *Akt2*-siRNA. The extent of HK-2 cell apoptosis, induced by exposure to high glucose medium (HG) for 24 h to mimic T1D, was assessed using flow cytometry (Figure 2B, C). The number of apoptotic cells was significantly higher in the HG-treated Con-siRNA group, but this effect was largely inhibited by FF treatment (*p* < 0.05). However, the reduction in HG-induced apoptotic cell death induced by FF in the *Akt2*-siRNA/HG/FF group was significantly less marked in the Con-siRNA/HG/FF group. This implies that FF inhibits HG-induced HK-2 cell apoptosis, which is partially Akt2-dependent.

### The impact of Akt2 silencing on FF-activated Nrf2 activity in HK-2 cells maintained in a HG or normal glucose environment

**Figure 3:**
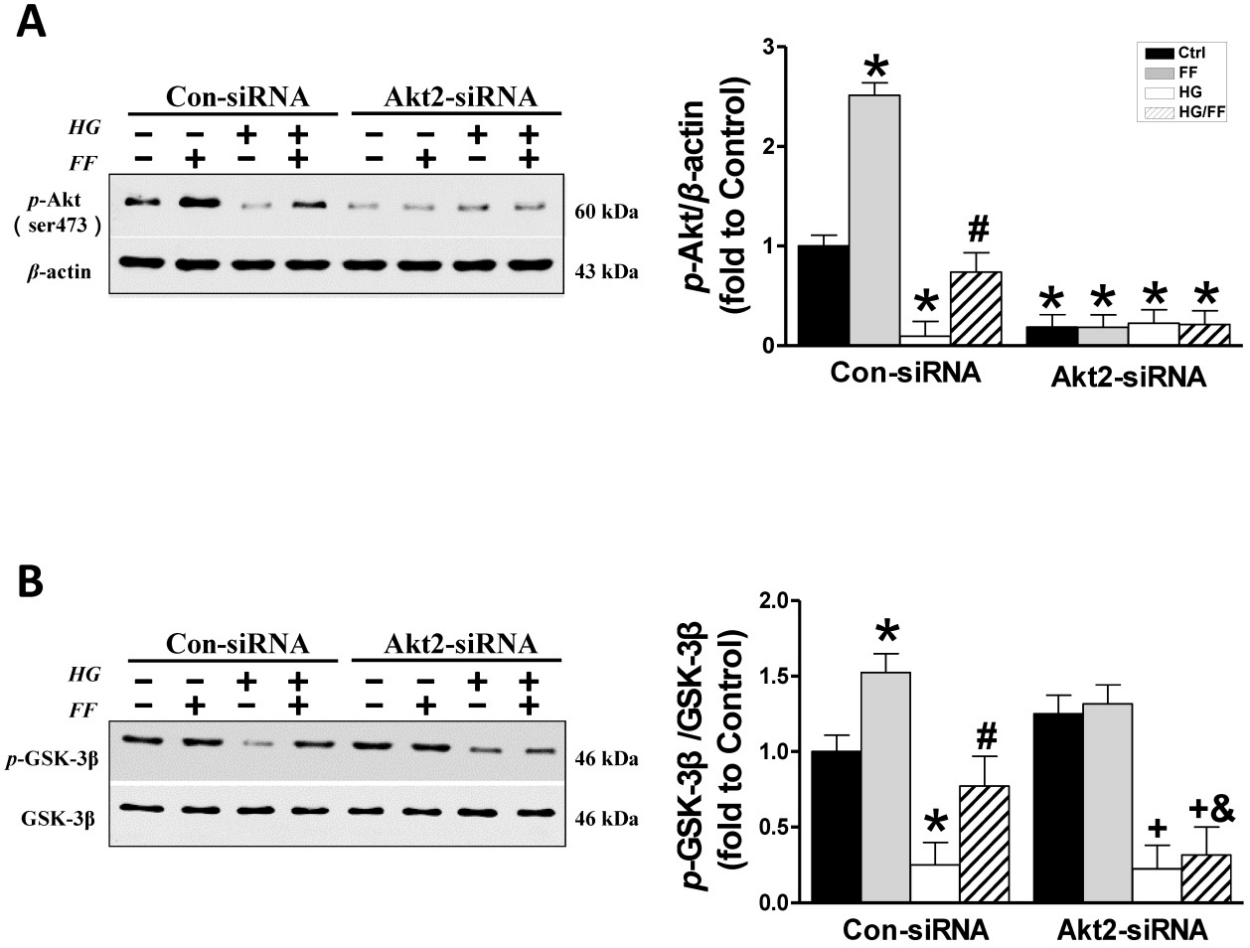
** The role of Akt2 in the FF-induced protection of HK-2 cells against HG.** Western blotting was used to measure Akt phosphorylation (A), and total and phosphorylated GSK-3β expression (B). Data were collected from at least three independent experiments and are presented as mean ± SD (n≥5). *, *p* < 0.05 *vs*. the corresponding Con-siRNA/Ctrl; #, *p* < 0.05 *vs*. the corresponding Con-siRNA/HG; &, *p* < 0.05 *vs*. the corresponding Con-siRNA/HG/FF; +, *p* < 0.05 *vs*. the corresponding *Akt2*-siRNA/Ctrl.

Considering that FF protection from T1D-induced nephropathy relies on FGF21-dependent activation of Nrf2 function and the Akt/GSK-3β/Fyn signaling pathway 19, we evaluated the impact of silencing Akt2 expression on FF-induced Nrf2 activity in HK-2 cells maintained in a HG or normal glucose environment. Phosphorylation of GSK-3β inactivates this kinase, such that it cannot phosphorylate Fyn (a negative regulator of Nrf2) and cause it to enter the nucleus, resulting in the export of Nrf2 from the nucleus. Therefore, we first determined the effect of FF on the Akt/GSK-3β/Fyn signaling pathway in *Akt2*-siRNA-transfected HK-2 cells. Western blotting of phosphorylated Akt showed that in Con-siRNA cells FF significantly increased, while exposure to HG significantly decreased the level of Akt phosphorylation (*p* < 0.05, Figure 3A). However, treatment with FF prevented the HG-induced reduction in *p*-Akt. Silencing of Akt2 inhibited both Akt phosphorylation (Figure 3A) and Akt2 expression (Figure 2A).

As shown in Figure 3B, FF significantly increased and HG significantly decreased GSK-3β phosphorylation (*p* < 0.05) in Con-siRNA groups, and this HG-induced inhibition of GSK-3β phosphorylation was partially prevented in Con-siRNA/HG/FF HK-2 cells. However, in *Akt2*-siRNA-transfected HK-2 cells, FF neither increased GSK-3β phosphorylation in control cells, nor prevented the HG-induced decrease in GSK-3β phosphorylation (Figure 3B).

**Figure 4:**
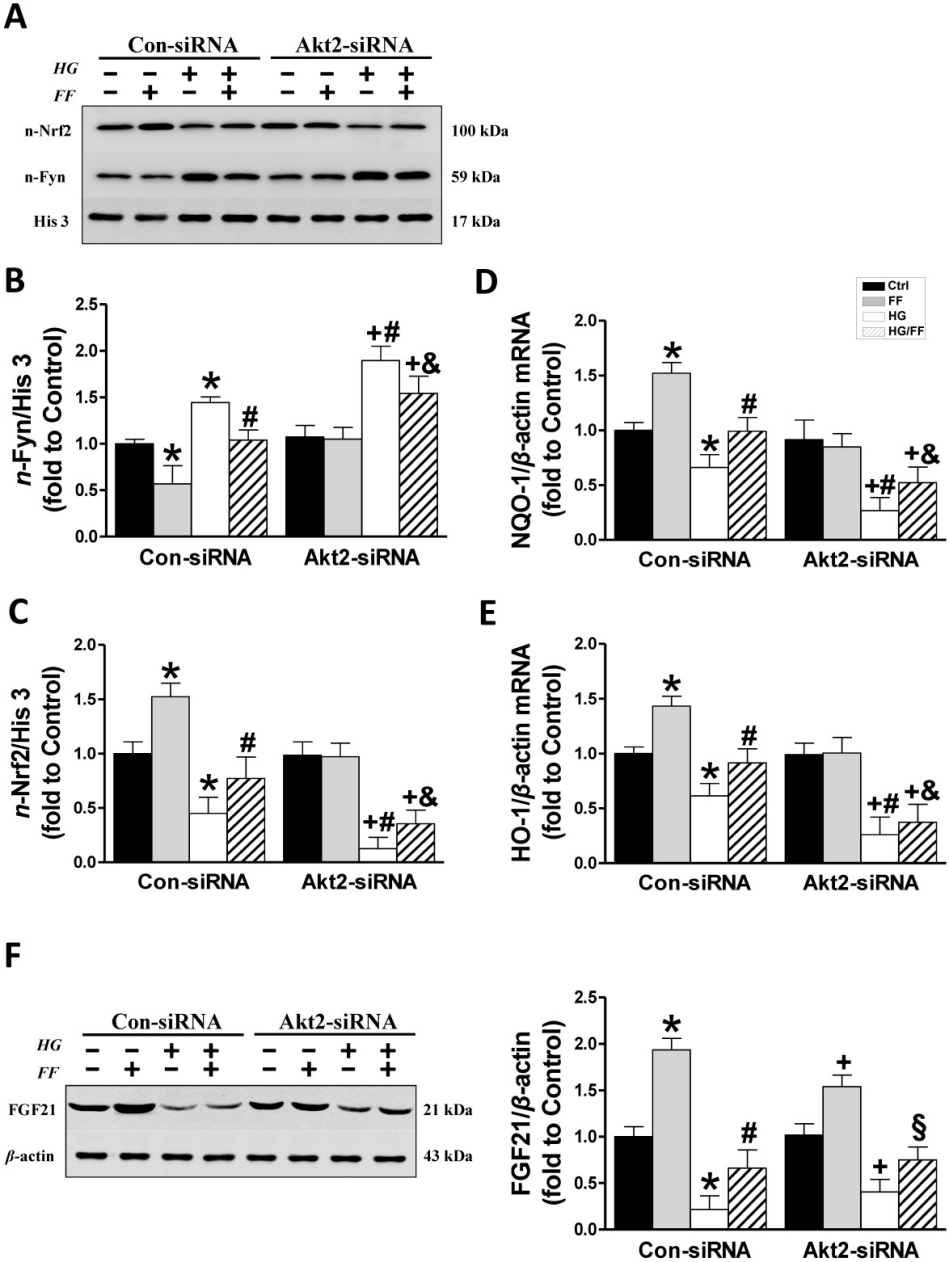
** Downstream mediators of the FF-induced protection of HK-2 cells against HG.** The expression of nuclear Fyn (AB) and Nrf2 (AC) was measured by western blotting. The transcriptional activity of Nrf2 was assessed by measuring the expression of its target genes, *NQO-1* (D) and *HO-1* (E), by qRT-PCR. (F) FGF21 expression. Data were collected from at least three independent experiments and are presented as mean ± SD (n≥5). *, *p* < 0.05 *vs*. the corresponding Con-siRNA/Ctrl; #, *p* < 0.05 *vs*. the corresponding Con-siRNA/HG; &, *p* < 0.05 *vs*. the corresponding Con-siRNA/HG/FF; +, *p* < 0.05 *vs*. the corresponding *Akt2*-siRNA/Ctrl; §, *p* < 0.05 *vs*. the corresponding *Akt2*-siRNA/HG.

Next, the subcellular distributions of Fyn and Nrf2 were assessed by western blotting in cells exposed to the various treatments (Figure 4A-C). Nuclear accumulation of Fyn was increased in Con-siRNA/HG cells and further significantly increased in *Akt2*-siRNA/HG cells (*p* < 0.05). FF significantly reduced nuclear Fyn levels in Con-siRNA groups, whether the cells were kept in HG or normal glucose concentrations, compared with control and HG-treated cells alone (*p* < 0.05). In *Akt2*-siRNA cells, HG caused a marked increase in nuclear Fyn accumulation compared with Con-siRNA/HG cells, but this was not prevented by FF treatment (Figure 4A, B).

In Con-siRNA-transfected cells, FF significantly increased, but HG significantly reduced, nuclear Nrf2 expression (*p* < 0.05), and its expression level in HG-treated cells was significantly lower than in control cells (*p* < 0.05), while nuclear Nrf2 expression in HK-2 cells treated with FF (HG/FF) was significantly higher (*p* < 0.05). In *Akt2*-siRNA-transfected HG-treated cells, nuclear Nrf2 expression was significantly lower than in cells kept in normal glucose medium (*p* < 0.05), and Nrf2 expression could not be stimulated by FF treatment in cells kept either in HG or normal glucose medium (Figure 4A, C).

To further confirm that Nrf2 is activated by FF, the mRNA expression of downstream gene targets of Nrf2 was measured using qRT-PCR. As shown in Figure 4D and E, mRNA expression of *NQO-1* and *HO-1* was significantly decreased in Con-siRNA/HG HK-2 cells, and further decreased in *Akt2*-siRNA/HG cells. However, when the cells were kept in normal glucose medium, FF stimulated the expression of both *NQO-1* and *HO-1* in Con-siRNA cells, but not in *Akt2*-siRNA cells. By contrast, FF significantly increased *NQO-1* and *HO-1* expression in Con-siRNA/HG/FF cells, but not in *Akt2*-siRNA/HG/FF cells. These results indicate that FF activates Nrf2 predominantly through the Akt2/GSK-3β/Fyn signaling pathway.

### Expression of Akt2 does not affect FF-induced FGF21 expression in HK-2 cells

Our previous study 19showed that FF treatment significantly protected by T1D mice from DN and FF-induced renal Akt phosphorylation was fully FGF21-dependent, suggesting that Akt2 activation by FF might be a downstream effect of FGF21. To confirm this, we measured FGF21 expression in HK-2 cells by western blotting (Figure 4F). HG significantly reduced FGF21 expression in Con-siRNA cells (*p* < 0.05), but FF treatment significantly ameliorated this effect (*p* < 0.05). In *Akt2*-siRNA-treated cells, the same FGF21 expression trend was identified, with FF increasing its expression in *Akt2*-siRNA-treated cells in a HG environment (*p* < 0.05), indicating that the silencing of Akt2 has no impact on FGF21 expression.

### The effects of FF on STZ-induced diabetes in Akt2-KO and WT mice

**Figure 5:**
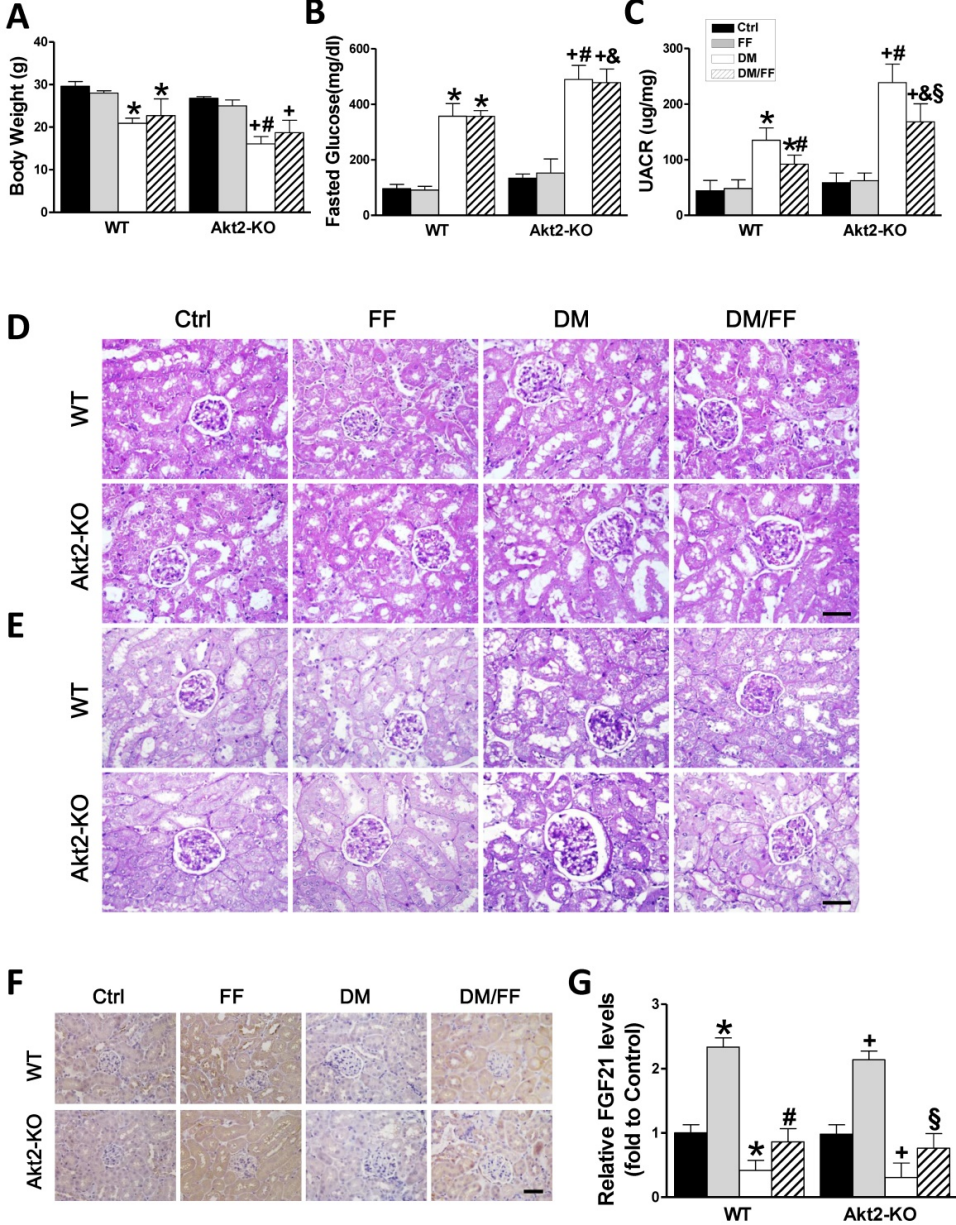
** Effects of FF on *Akt2*-KO and WT mice with diabetes.** Diabetes was induced by a single intraperitoneal injection of streptozotocin (STZ, 150 mg/kg), and then mice were administered FF (100 mg/kg) or vehicle by gavage every other day for 3 months. Body mass (A), fasting glucose (B), and urinary albumin-to-creatinine ratio (UACR, C) were measured or calculated at the end of the treatment period. Renal histology (D) and glycogen deposition (purple) (E) were assessed following hematoxylin and eosin (H&E) and periodic acid-Schiff (PAS) staining of kidney sections (400 ×, scale bar 100 μm), respectively. Immunohistochemistry (IHC) for fibroblast growth factor 21 (FGF21) (F; brown: positive staining) was performed in kidney sections (400 ×, scale bar 100 μm), followed by quantitative analysis (G). Data are presented as the mean ± SD (n≥5). *, *p* < 0.05 *vs*. the corresponding WT/Ctrl; #, *p* < 0.05 *vs*. the corresponding WT/DM; &, *p* < 0.05 *vs*. the corresponding WT/DM/FF; +, *p* < 0.05 *vs*. the corresponding *Akt2*-KO/Ctrl; §, *p* < 0.05 *vs*. the corresponding *Akt2*-KO/DM.

To confirm the results of the vitro studies, we induced diabetes using STZ in both *Akt2*-KO and WT mice. Both *Akt2*-KO and WT diabetic mice were treated with FF for 3 months after the onset of diabetes, and body mass and blood glucose were measured. As shown in Figure 5A and B, the body mass increase in WT diabetic mice was significantly lower than that in WT control mice (*p* < 0.05), and still lower in *Akt2*-KO diabetic mice (*p* <0.05). In the diabetic mice, FF treatment had a slight or no effect on mass gain in both WT and *Akt2*-KO mice (*p* > 0.05). The fasting blood glucose concentrations of WT and *Akt2*-KO diabetic mice were significantly higher than that of control mice, and that of the *Akt2*-KO/DM group was higher than that of the WT/DM group (*p* < 0.05). However, FF treatment had no significant effect on blood glucose.

Renal function, assessed using the UACR (Figure 5C), was not significantly different between non-diabetic *Akt2*-KO and WT mice (*p* > 0.05), but the UACR of *Akt2*-KO/DM mice was significantly higher than that of WT/DM mice (*p* < 0.05), and FF treatment partially corrected this (*p* < 0.05). H&E staining showed that, compared with the control group, glomerular volume was higher (Figure 5D), the glomerular basement membrane was irregularly thickened, the mesangial and extracellular matrix was hyperplastic, the tubular lumen was dilated (Figure 5D), and renal glycogen deposition was more marked (Figure 5E) in WT/DM mice, and these pathologic changes were worse in *Akt2*-KO/DM mice. FF treatment significantly ameliorated the renal pathology in WT/DM/FF mice, but only partially ameliorated the renal pathology in *Akt2*-KO/DM/FF mice.

Next, the expression of FGF21 was examined by IHC staining (Figure 5F, G) showed that renal FGF21 expression was significantly lower in both the WT/DM and *Akt2*-KO/DM groups (*p* < 0.05), but was similar to the control level in both WT/DM/FF and *Akt2*-KO/DM/FF mice treated with FF. Therefore, deletion of the *Akt2* gene does not affect the upregulation of FGF21 in response to FF.

### Akt2 gene deletion partially prevents the FF-mediated renal protection in STZ-induced diabetes mice

**Figure 6:**
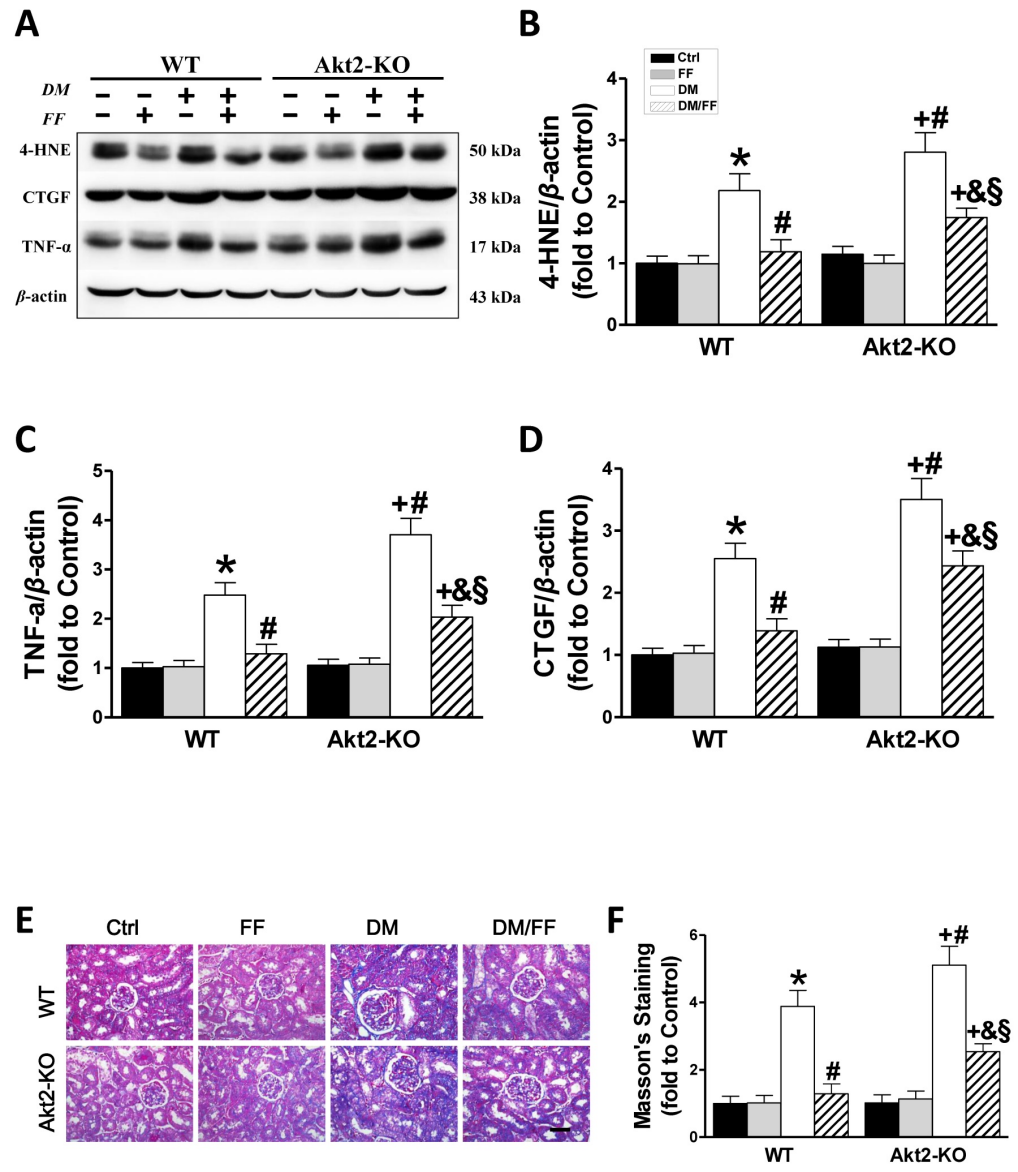
** Akt2 gene deletion partially abolishes the protective effect of FF against diabetes-associated renal damage.** Renal oxidative damage was evaluated by western blotting for 4-hydroxy-2-nonenal (4-HNE) (AB). Renal inflammation was identified by western blotting for tumor necrosis factor-α (TNF-α) (AC). The severity of the renal fibrotic response was reflected in greater expression of connective tissue growth factor (CTGF), demonstrated using western blotting (AD). Masson's staining (E) was used to detect collagen fibers (blue) in kidney sections (400 ×, scale bar 100 μm). Semi-quantitative data generated from the Masson's-stained sections are presented in F. Data are presented as the mean ± SD (n≥5). *, *p* < 0.05 *vs*. the corresponding WT/Ctrl; #, *p* < 0.05 *vs*. the corresponding WT/DM; &, *p* < 0.05 *vs*. the corresponding WT/DM/FF; +, *p* < 0.05 *vs*. the corresponding *Akt2*-KO/Ctrl; §, *p* < 0.05 *vs*. the corresponding *Akt2*-KO/DM.

Western blotting for 4-HNE (Figure 6A, B) and TNF-α (Figure 6A, C) showed that in both WT and *Akt2*-KO mice the degree of renal oxidative stress and the inflammatory response in diabetic mice were significantly higher than those of non-diabetic mice (*p* < 0.05), but worse in *Akt2*-KO/DM than WT/DM mice (*p* < 0.05). FF treatment almost completely eliminated the diabetes-associated oxidative stress and inflammation in FF-treated WT/DM/FF mice, but had a significantly smaller effect in *Akt2*-KO/DM/FF mice.

Western blotting for CTGF, a pro-fibrotic mediator (Figure 6A, D), showed that renal CTGF expression was high in WT/DM mice and higher still in *Akt2*-KO/DM mice (*p* <0.05). FF treatment completely prevented the diabetes-induced increase in CTGF expression in WT/DM/FF mice, but had a smaller effect in *Akt2*-KO/DM/FF mice. The pro-fibrotic effect of diabetes in the kidneys was also demonstrated by Masson's staining (Figure 6E), followed by semi-quantitative analysis (Figure 6F), which showed the same trend as CTGF expression. These findings confirm that *Akt2* is required for full protection against the diabetes-associated renal oxidative stress, inflammation, and fibrosis that is induced by FF.

### An AMPK inhibitor partially blocks FF-mediated improvement of kidney injury in STZ-induced diabetes mice

**Figure 7:**
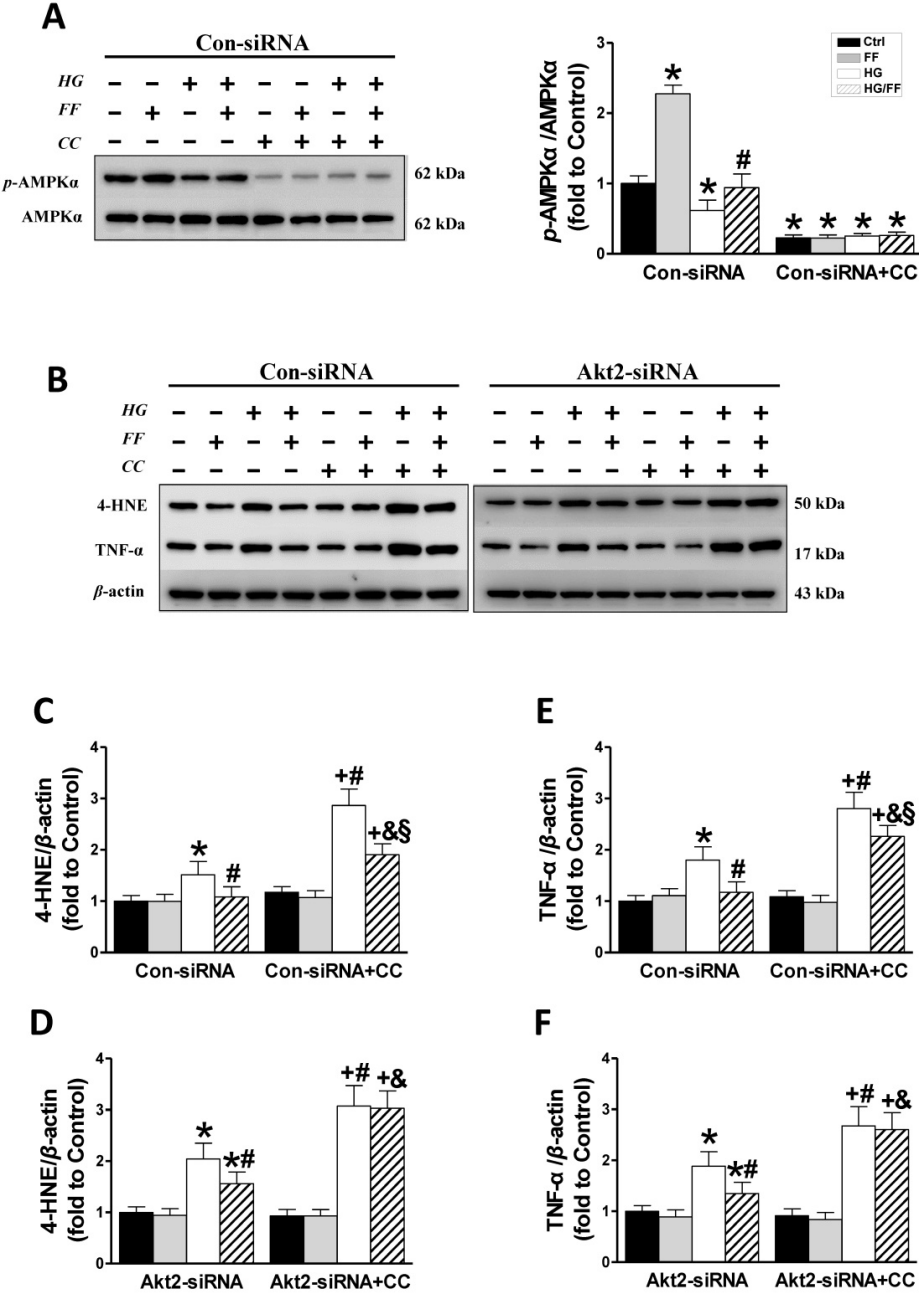
** The role of AMPK in the FF-induced protection of HK-2 cells against HG.** HK-2 cells were treated with either the control or Akt2-specific siRNA, and then the cells were treated with combinations of HG, FF and AMPK inhibitor (compound C, CC) for 24 h. Western blotting was used to measure total and phosphorylated AMPK expression (A). Renal oxidative damage was evaluated by western blotting for 4-HNE (BC, BD). Renal inflammation was detected by western blotting for TNF-α (BE, BF). Data were collected from at least three independent experiments and are presented as mean ± SD (n≥5). *, *p* < 0.05 *vs*. the corresponding Con-siRNA/Ctrl or *Akt2*-siRNA/Ctrl; #, *p* < 0.05 *vs*. the corresponding Con-siRNA/HG or* Akt2*-siRNA/HG; &, *p* < 0.05 *vs*. the corresponding Con-siRNA/HG/FF or *Akt2*-siRNA/HG/FF; +, *p* < 0.05 *vs*. the corresponding Con-siRNA+CC/Ctrl or *Akt2*-siRNA+CC/Ctrl; §, *p* < 0.05 *vs*. the corresponding Con-siRNA+CC/HG.

Western blotting of phosphorylated AMPKα (Figure 7A) showed that in Con-siRNA cells FF significantly increased, while exposure to HG significantly decreased the level of AMPKα phosphorylation (*p* < 0.05). However, treatment with FF prevented the HG-induced decrease in *p*-AMPKα, and AMPK inhibitors (compound C, CC) significantly inhibited AMPKα phosphorylation in Con-siRNA cells (Figure 7A).

Western blotting for renal oxidative stress 4-HNE (Figure 7B, C) showed that 4-HNE expression increased when cells were exposed to HG and increased further when they were exposed to HG and the AMPK inhibitor (*p* <0.05). FF treatment completely prevented the HG-induced increase in 4-HNE expression in Con-siRNA cells, but only partially prevented this increase in Con-siRNA+CC cells. In *Akt2*-siRNA cells, 4-HNE expression (Figure 7B, D) increased significantly in HG-treated cells, but this increase was inhibited by FF. Treatment with the AMPK inhibitor resulted in a sharp increase in 4-HNE expression (*p* <0.05), but this increase was not affected by FF.

The renal inflammatory response was evaluated by western blotting for TNF-α. As shown in Figure 7B, E and F, TNF-α expression was higher in Con-siRNA and *Akt2*-siRNA cells exposed to HG. In Con-siRNA cells, the AMPK inhibitor and FF partially reduced TNF-α expression, whereas in *Akt2*-siRNA cells, FF did not decrease TNF-α expression after the addition of the AMPK inhibitor. These results indicate that the AMPK signaling pathway is partially responsible for FGF21-mediated renal protection against a high glucose environment.

## Discussion

**Figure 8:**
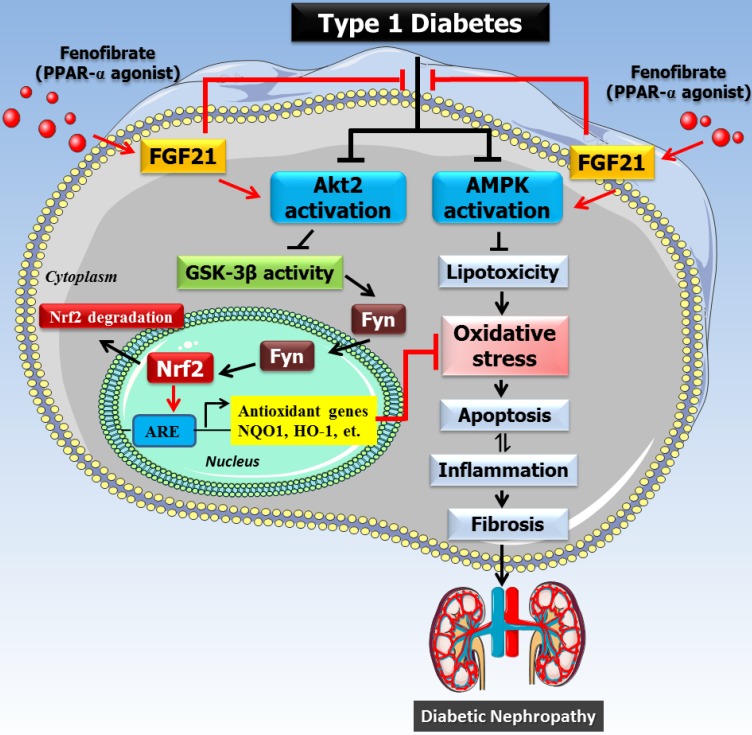
** The mechanism of the protective effect of fenofibrate against diabetic nephropathy.** Oxidative stress is a key pathogenic factor in the development of DN. Under diabetic conditions, glucose metabolism is impaired and excessive amounts of reactive oxygen species (ROS) are produced, which accumulate in the kidney and induce oxidative stress. Oxidative stress causes renal cellular injury and apoptosis, followed by inflammation and fibrosis, which ultimately leads to renal dysfunction. Our previous study showed that FF-induced renal protection from diabetes is mediated through an upregulation of FGF21, and this in turn stimulates Akt/GSK-3β/Fyn-mediated activation of the Nrf2 anti-oxidative pathway. The present study reveals that the protective effect of FF/FGF21 on DN is dependent on Akt2. The activation of the Akt2/GSK-3β/Fyn pathway increases the nuclear translocation of Nrf2 by inhibiting the nuclear accumulation of Fyn, and induces antioxidant gene expression. However, AMPK activation can also alleviate the metabolic defects, and both pathways prevent renal oxidative stress, inflammation, and remodeling.

In our previous study, we showed that the PPARα agonist FF prevents T1D-induced renal oxidative stress, cell apoptosis, inflammation, fibrosis, and dysfunction 19, and that this is associated with an increase in Nrf2 activation. Here, we have shown that the FF-induced upregulation of Nrf2 activity is predominantly dependent on the Akt2/GSK-3β/Fyn pathway. Our previous study also showed that the renal protective effect of FF in diabetes is FGF21-dependent, and here we have further confirmed that FGF21 is upstream of the Akt2/GSK-3β/Fyn/Nrf2 pathway, as illustrated in Figure 8, because deletion of Akt2 *in vitro* (Figure 4F) and *in vivo* (Figure 5G) does not affect FF-induced FGF21 expression. In the present study, we further reveal for the first time that FF upregulates the expression of FGF21 in the kidney of T1D mice. In turn, FGF21 not only activates the Akt2-dependent oxidative response under the control of Nrf2, but also activates AMPK-dependent metabolic processes to the nucleus, where it upregulates the transcription of downstream antioxidant molecules that protect against T1D-induced renal oxidative stress/damage, inflammation, fibrosis, and consequently dysfunction. Because partial renal protection by FF (Figures 5C, 6) remained in *Akt2*-KO mice, we further showed that FF also stimulates AMP-activated protein kinase (AMPK)-mediated renal protection in T1D-associated DN (Figures 7). In addition, although we showed that AMPKα was activated using a phospho-specific antibody, but we were unable to conclude that FF-activated renal protection was AMPKα-dependent because we did not examine other AMPK isoforms, although in previous reports describing FF activation of AMPK 25-27, only couple of them directly or indirectly measured AMPKα induction by FF and hardly any mentioned AMPK isoforms 25-27 .

The protein kinase Akt, also known as protein kinase B (PKB), has been shown to regulate a variety of cell functions, and is particularly important for glucose metabolism, cell growth, and cell survival. Therefore, changes in its expression or activity are thought to be involved in the pathogenesis of diabetes and DN 28. In mammals, there are three isoforms of Akt: Akt1 (PKBα), Akt2 (PKBβ), and Akt3 (PKBγ), which each have their own physiologic functions. Akt1 is involved in cell survival and protein synthesis, inhibiting apoptosis, whereas Akt2 is an important regulator of metabolism. The specific function of Akt3 remains unclear, but it may be involved in the development and size of the brain 29. Kim *et al*. measured the expression of all three isoforms of Akt in obese rats and found very low expression and activity of Akt3 in insulin-sensitive tissues. There were no significant differences in the amount of Akt1 protein in tissues of obese and lean rats, whereas there was 56% less Akt2 in muscle and 35% less in the liver of obese rats 30. Similarly, we found that the phosphorylation of both Akt1 (Figure 1A, B) and Akt2 was lower in the kidney of T1D mice, and the expression of Akt2 was also significantly lower. FF upregulated the phosphorylation of Akt2 in diabetic mice (Figure 1A, C), but there were no significant differences in the renal expression of Akt3 among the groups (Figure 1A, D).

The development of DN has multiple aspects to its pathogenesis, including apoptosis, oxidative stress, inflammation, and fibrosis. Therefore, the precise role of Akt2 in the development of DN in the present study may differ from that involved in the mediation of the TGF-β effect in HK-2 cells 28. In the pathogenesis of DN, the metabolic disturbance and the associated oxidative stress have been considered to be the principal mechanisms responsible. We and others have shown the preventive effect of FF treatment against DN 19 and diabetic retinopathy 31, which occurs concomitantly with an increase in Nrf2 activity, implying that oxidative stress plays a role in the pathogenesis of diabetic complications. In our previous study, we showed that FGF21 is essential for FF-mediated Nrf2 activation and renal protection. It is known that FGF21 is an important regulator of energy metabolism and has anti-oxidative effects 15-17. Ye *et al.* have shown that FGF21 upregulates the expression of Nrf2 15, and another study showed that FGF21 protects against oxidative damage in the liver by activating both Nrf2 and Akt signaling 17 and against the diabetes-associated apoptosis of germ cells 18.

Rizvi *et al*. showed that the antioxidant capacity of the Nrf2/antioxidant responsive element (ARE) pathway could be upregulated *via* the Akt/GSK-3β/Fyn axis 32. Therefore, we determined the effect of Akt2 deletion on the activation of the Akt/GSK-3β/Fyn signaling pathway and the Nrf2 antioxidant pathway. Both the *in vitro* data (Figure 4F) and IHC staining (Figure 5F, G) showed that renal FGF21 expression was not influenced by *Akt2* deletion, suggesting that Akt2 is a downstream mediator of the effects of FGF21. Then, using cultured human renal tubular epithelial cells, we demonstrated that the Akt2/GSK-3β/Fyn signaling pathway is an important regulator of Nrf2 activation. Therefore, we proposed that FF-induced FGF21 expression, mediated *via* PPARα, could protect the kidney from diabetes-induced oxidative stress/damage, inflammation, and remodeling by activating the Akt2/GSK-3β/Fyn/Nrf2 pathway, similar to the mechanism reported to mediate the hepatoprotective effect of the Akt/Nrf2 pathway in diabetes 32. However, the fact that deletion of Akt2 only partially inhibited FF/FGF21-induced renal protection (Figure 5C and Figure 6) suggests that another mechanism may be responsible for the renal protective effect of FF/FGF21 in DN.

To determine whether the increase in phosphorylation of Akt2 induced by FF may play a pivotal role in the protective effect of FF against DN, we studied *Akt2*-KO and WT mice in which diabetes had been induced by STZ injection. In this way, we were able to define an important, but not exclusive, role for Akt2 in the FF-induced protection against DN. Akt2 is expressed in multiple organs, including the heart, brain, small intestine, and kidney. Mice deficient in Akt2 develop hyperglycemia, hyperinsulinemia, insulin resistance, age-dependent loss of adipose tissue, and diabetes in males 33, 34. Koren *et al.* found that *Akt2*-KO mice grow normally but develop moderate hyperglycemia, and the liver and skeletal muscle of these mice show poorer responses to insulin than normal mice 35. However, the body mass of *Akt2*-KO/DM mice was significantly lower than that of control mice of the same age, and their blood glucose concentrations were significantly higher than those of the control group, which may be due to both the T1D and T2D-like syndrome in *Akt2*-KO/DM mice. It has been reported that deletion of the *Akt2* gene causes loss of the normal skeletal structure of glomerular podocytes, leading to the apoptosis and fusion of podocytes, which results in severe proteinuria and glomerular sclerosis 36. Therefore, we undertook H&E and PAS staining to evaluate these changes, and found that the renal pathology was worse in *Akt2*-KO/DM than WT/DM mice (Figure 5D, E). FF treatment had no significant effect on body mass or blood glucose, but almost completely prevented the development of DN in WT diabetic mice, and partially prevented the renal hypertrophy and histologic changes in *Akt2*-KO diabetic mice (Figure 6). This suggests that although Akt2-mediated protective mechanisms play an important role, there is also an Akt2-independent mechanism for the renal protective effects of FF in DM.

The severity of renal oxidative stress, inflammation, apoptosis, and fibrosis represent indices of renal damage. Under conditions of oxidative stress, the major responses are cell death and the activation of Akt signaling 37, 38. Zhang *et al*. has demonstrated that Akt2 is important for the prevention of oxidative damage 39. They found that oxidative stress significantly upregulates Akt2 expression and total Akt activity, which affects the activity of its downstream targets and thereby prevents oxidative stress-induced apoptosis. In the present study, the levels of oxidative stress (Figure 6A, B) and inflammation (Figure 6A, C) in the kidneys of *Akt2*-KO/DM mice were more severe than in WT/DM mice, and FF treatment could only slightly ameliorate the oxidative stress and inflammatory response in the absence of Akt2. CTGF (Figure 6A, D) protein and Masson's staining of kidney sections (Figure 6E, F) were used to evaluate the degree of renal fibrosis in diabetic mice, alongside the assessment of collagen deposition and the measurement of fibrotic protein expression. However, our findings were contrary to those of a previous *in vitro* study, which showed that TGF-β1 treatment enhances Akt2 expression in HK-2 cells and that deletion of the *Akt2* gene suppresses TGF-β1-induced epithelial-mesenchymal transition (EMT) 28, which is thought to contribute to renal fibrosis in DN. This suggested that Akt2 may be a pivotal mediator of EMT induced by TGF-β1.

However, based on the results mentioned above, the Akt2-mediated protective mechanism, although important, may only partially protect against DM; thus, it remains to be determined whether FF-mediated protection against DM also involves an Akt2-independent mechanism. The involvement of AMPK-mediated signaling in the protective effect of FGF21 in various organs during diabetes or other diseases has been reported 18, 40-42. In the current study (Figure 8), we propose that AMPK is partially responsible for the FGF21-mediated renal protection from HG because the use of an AMPK inhibitor alongside Akt2-siRNA silencing abolished the preventive effect of FF on the HG-induced phenotype of HK-2 cells. In fact, Al-Rasheed *et al*. have previously described the involvement of the activation of liver kinase B1/AMPK in the FF-induced protection against DN in T1D rats 27. Although we and others have not determined how FGF21 activates AMPK in T1D mice and rats 27, there is convincing evidence that FGF21 activates the AMPK pathway, either directly through fibroblast growth factor receptor 1/β-klotho signaling, or indirectly by stimulating the secretion of adiponectin and corticosteroids, which can activate AMPK signaling in their target tissues 43. Therefore, FF protects against T1D-induced DN *via* a PPARα-mediated increase in FGF21, which activates Akt2/GSK-3β/Fyn/Nrf2 signaling to upregulate the Nrf2-mediated antioxidant mechanism, but also *via* activation of AMPK, which mediates an amelioration of the metabolic defects. However, the activation of both pathways results in the prevention of renal oxidative stress, inflammation, and remodeling, as outlined in Figure 8.

In summary, the present study has, for the first time, identified an essential role of Akt2 in the FF/FGF21-induced renal protection in T1D, which is achieved *via* the activation of an Nrf2-mediated antioxidant mechanism. However, when these findings are combined with those of our *in vitro* experiments and of a previous study of T1D rats, we can conclude that AMPK also plays an important role in this mechanism. These two mechanisms together fully explain the renal protection from T1D induced by FF. This is highly significant because FF is safe and currently used in the clinic, and the identification of these mechanisms should significantly accelerate its adoption for the prevention of DN in diabetic patients.

## Abbreviations

4-HNE: 4-hydroxy-2-nonenal
AMPK: adenosine monophosphate-activated protein kinase
CC: compound C
CTGF: connective tissue growth factor
DN: diabetic nephropathy
FF: fenofibrate
FGF21: fibroblast growth factor 21
HK-2: human renal tubular epithelial cells
Nrf2: nuclear factor erythroid 2-related factor 2
PPARα: peroxisome proliferator-activated receptor-alpha
p-Akt: phosphorylated Akt
t-Akt: total Akt
p-GSK-3β: phosphorylated glycogen synthase kinase-3β
t-GSK-3β: total glycogen synthase kinase-3β
TNF-α: tumor necrosis factor-alpha
T1D: type 1 diabetic
TGF-β1: transforming growth factor-β1
UACR: urinary albumin-to-creatinine ratio
